# Identifying large-scale patterns of unpredictability and response to insolation in atmospheric data

**DOI:** 10.1038/srep45676

**Published:** 2017-03-30

**Authors:** Fernando Arizmendi, Marcelo Barreiro, Cristina Masoller

**Affiliations:** 1 Instituto de Física, Facultad de Ciencias, Universidad de la República, Iguá 4225, Montevideo, Uruguay; 2 Departament de Física, Universitat Politecnica de Catalunya, 08222 Terrassa, Barcelona, Spain

## Abstract

Understanding the complex dynamics of the atmosphere is of paramount interest due to its impact in the entire climate system and in human society. Here we focus on identifying, from data, the geographical regions which have similar atmospheric properties. We study surface air temperature (SAT) time series with monthly resolution, recorded at a regular grid covering the Earth surface. We consider two datasets: NCEP CDAS1 and ERA Interim reanalysis. We show that two surprisingly simple measures are able to extract meaningful information: i) the distance between the lagged SAT and the incoming solar radiation and ii) the Shannon entropy of SAT and SAT anomalies. The distance uncovers well-defined spatial patterns formed by regions with similar SAT response to solar forcing while the entropy uncovers regions with similar degree of SAT unpredictability. The entropy analysis also allows identifying regions in which SAT has extreme values. Importantly, we uncover differences between the two datasets which are due to the presence of extreme values in one dataset but not in the other. Our results indicate that the distance and entropy measures can be valuable tools for the study of other climatological variables, for anomaly detection and for performing model inter-comparisons.

Large-scale climate phenomena have attracted great interest in the last decades, as improving the understanding of climate interactions is crucial for advancing long-term forecasts. A lot of research is nowadays focused in the development of appropriated data analysis tools, and a methodology that has been proven to be valuable is based on complex networks^1^,^2^,^3^,^4^. Within this approach, a climate network is defined over a regular grid of geographical locations (nodes), and the links between pairs of nodes are defined by performing bi-variate analysis of the time series of climate variables recorded at the nodes. Different measures have been used to infer the links (cross-correlation, the mutual information, the conditional mutual entropy, Granger causality^5^,^6^,^7^,^8^,^9^,^10^,^11^,^12^,^13^), and similar, or different, network structures have been unveiled, depending on the measure employed and the significance confidence level used.

Important challenges for inferring the connectivity of the climate system include the role of external solar forcing and the role of climate variability with different time-scale with respect to that of the analysis. For example, when the analysis is focused on phenomena at inter-annual or longer time-scales, weather systems concentrated at 3–7 days may be regarded as noise.

A potential drawback of the climate network method is that, if the time series in two nodes have similar characteristics, these regions can appear as “linked”, in spite of the fact that there might not be genuine underlying interaction between the climate variables in the two regions. Climatic similarities can be due to physical processes that act in a similar way in distant regions, and produce similar effects. Such climatic similarities might be reflected as network links, depending on the similarity measure used to construct the network^13^. On the other hand, regions with different climate characteristics may as well be linked by genuine long-range interactions. It is therefore important to perform uni-variate time series analysis to yield insight into the interpretation of the inferred links.

Here we consider two reanalysis datasets (NCEP CDAS1 and ERA Interim) and analyze the properties of surface air temperature (SAT) time series with monthly resolution, focusing on quantifying the SAT response to solar forcing and the degree of disorder or unpredictability of SAT variability. We chose the SAT field because it is an important climatological field that has been commonly used to define climate networks^2^,^3^,^4^,^5^,^6^,^7^,^9^,^11^. We address the following questions: is it possible to identify geographical regions with similar SAT response to solar forcing? Where are the regions with strongest distortion? Is it possible to identify regions with similar degree of SAT unpredictability? Where are the most unpredictable regions? We are interested in the relationship between solar forcing and SAT unpredictability because is important to characterize the geographical regions where this field is more/less predictable. A main contribution to SAT predictability comes from solar forcing that induces, in many regions, an oscillatory behavior with a well-defined periodicity. In hydrology (stream flow daily data), the effect of elimination of seasonality leads to much more random behavior^14^.

To study SAT response to solar forcing we use the insolation (i.e., the local top-of-atmosphere incoming solar radiation) as a proxy of solar forcing and compare the SAT and the insolation waveforms by means of a distance measure between time series. As we are interested in assessing the similarity of the two waveforms, the two time series are normalized to zero mean and unit variance and the SAT time series (response) is shifted by an appropriated number of months, *τ* > 0, with respect to the insolation (forcing). To quantify the degree of unpredictability or disorder of SAT time series we use the standard measure of information theory, Shannon entropy^15^, computed from the probability distribution function (PDF) of SAT and SAT anomaly values (the anomalies are calculated from SAT time series by removing the seasonal cycle).

The distance and entropy measures employed here are well known. A drawback of the distance measure is that it is non-zero for general forms of linear convolutions [i.e., it returns an non-zero value for the distance between *x* and *y*, if they are linearly related as 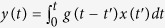]. In spite of this drawback, our results demonstrate that, at least for the analysis of monthly SAT time series, the distance yields meaningful results. Regarding the entropy measure, a main drawback is that it does not capture the information about the ordering of the data values in the time series: the entropy returns the same value if the data is shuffled randomly. This is because randomly shuffling the data not modify the PDF of SAT values. In spite of these important drawbacks, we demonstrate that both, the distance and the entropy provide meaningful information about large-scale atmospheric phenomena. Specifically, in tropical oceanic regions, well defined spatial patterns are uncovered, which have large distance and low entropy values; in the extra-tropics, localized regions associated with sea ice are detected; in the continents, rainforest regions are also detected. In addition, in specific geographical regions, differences between the two reanalysis dataset are identified, which are due to the presence of extreme values in the data (the quantitative definition of extreme values depends on the specific system–extreme fluctuations in hydrodynamics, climate or optics are of very different magnitude; however, a general criterion is that the PDF of the data values in the time-series has a non-Gaussian, long-tailed shape).

## Datasets and Measures

We consider monthly mean SAT data from two reanalysis data sets: NCEP CDAS1^16^ and ERA Interim^17^. The spatial resolution is 2.5°/1.5° and cover the time-period [1949–2015]/[1980–2014] respectively. The NCEP CDAS1 reanalysis has *N* = 10224 time series of length *L* = 792 while the ERA Interim, *N* = 28562 and *L* = 408. The insolation at the top of the atmosphere is calculated as in Berger^18^, as a function of day of year and latitude. Then, monthly averaged values for every latitude are calculated.

For each raw SAT time series, *r*_*i*_(*t*), we first normalize to zero-mean and unit variance, obtaining the normalized SAT, *y*_*i*_(*t*) [where 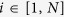 and 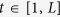]. Then, we calculate the *climatology* time series, *c*_*i*_(*t*), and the anomaly time series, *z*_*i*_(*t*) as follows. The climatology (or seasonal cycle) is computed as the SAT value each month, *y*_*i*_(*t*), averaged over the SAT values in that month in all years, 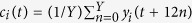, where *Y* = *L*/12 is the number of years (66 or 34 depending on the dataset). In this way we obtain a time series of length 12 months which is extended to cover the whole period 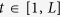 months by considering the climatology a periodic function with period equal to one year. The anomaly time series is the difference between the SAT and the climatology, i.e., *y*_*i*_(*t*) − *c*_*i*_(*t*).

To study SAT response to solar forcing we compute the distance between SAT time series in grid point *i*, {*y*_*i*_(*t*)}, and the insolation time series in the same point, {*x*_*i*_(*t*)}. The distance is calculated as


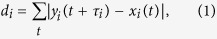


which is known as *taxicab* metric. In the Supplementary Information we demonstrate that similar results are obtained by using the Euclidean distance, 
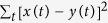
. Because we are interested in measuring the similarity of the shape of *x* and *y* waveforms, the two time series are normalized to zero mean and unit variance.

In Eq. (1) *τ*_*i*_ > 0 is a lag that allows to take into account inertia and/or memory effects, and needs to be appropriately chosen. This lag is expected to be more important in the oceans in comparison with land masses, because of the larger heat capacity of water. A natural choice is the value of *τ*_*i*_ that minimizes the distance between the insolation and the climatology (the averaged monthly SAT); similar results were found when considering the raw SAT time series instead. We search the minimum of the distance considering *τ*_*i*_ values in the interval of [0–4] months because of the lack of physical mechanisms that could result in a longer delayed response of the climatology to the insolation. The robustness of the results with respect to the maximum lag is shown in the Supplementary Information.

While SAT time-series has a strong deterministic component due to solar forcing (which imposes, in many regions a well-defined periodicity and thus, provides some predictability), SAT anomaly (SATA) values are much more unpredictable, because the deterministic part (the seasonal cycle) is essentially removed. To quantify the degree of disorder or unpredictability of both, SAT and SATA time series we calculate Shannon entropy^15^. The entropy is normalized to the maximum entropy, of the uniform distribution:


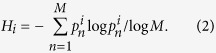


Here 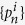, 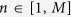 is the probability distribution of the values in the *i*th time series (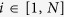) and *M* is the number of bins. *M* is the same for all time series within a reanalysis database, but is adjusted in each database to take into account the different length of the time series: we use *M* = 20 for ERA Interim and *M* = 40 for NCEP CDAS1. This gives similar average data points per bin (~20). In the Supplementary Information we demonstrate that similar results are obtained with lower *M*.

The bin size is determined by the local extreme values in each time series, i.e., 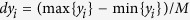 . While this choice, at first sight, might seem contradictory with performing “inter-site comparisons”, it allows to resolve with adequate precision the shape of the PDF in each site. The results of the entropy analysis when a uniform bin size is used (i.e., *dy* is computed from the global maximum and minimum values) are presented in the Supplementary Information. With a uniform bin size a good resolution of the shape of the PDF in each site is not possible because there are regions where SAT/SATA has large variations, and others in which the variations are considerably smaller.

## Results

**Figure 1:**
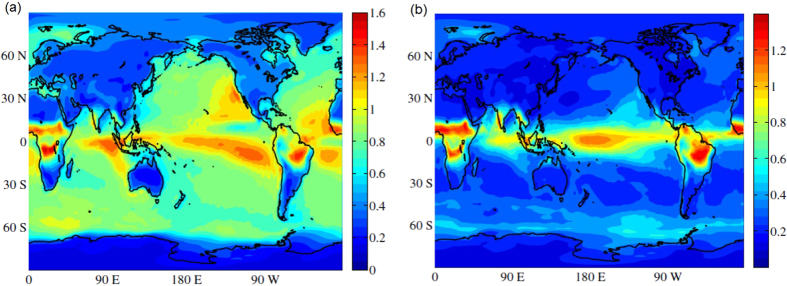
(**a**) Map of distances *d*_*i*_ calculated from Eq. (1) when the forcing (insolation) and the response (SAT) are not shifted (*τ*_*i*_ = 0). (**b**) Map of distances, when the forcing and the response are shifted *τ*_*i*_, with 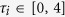. Matlab software (version number 7.12.0.635) was used to create these maps (https://www.mathworks.com/).

We begin by analyzing the distance between SAT and insolation time series. First, we consider the situation in which the SAT is not shifted with respect to the insolation, i.e., all lag times *τ*_*i*_ are taken equal to zero. Figure 1(a) displays the map of *d*_*i*_ values obtained from the analysis of the ERA Interim dataset. Similar results were obtained from the NCEP CDAS1 dataset (as shown in the Supplementary Information).

We observe that over continental areas the distance values are smaller than over the oceans, but there are exceptions, as in the Amazon and in the African rainforest, which show high *d*_*i*_ values. Well defined spatial structures are seen over the oceans, in the cold tongues and areas associated with easterly trades and upwelling processes.

The large difference between the *d*_*i*_ values in the oceans and in the continents is mainly due to the greater heat capacity of water, that results in long temperature memory in the oceans. When this effect is taken into account, at least in part, by shifting the SAT time series, the map of *d*_*i*_ values is strongly modified, as shown in Fig. 1(b). We observe that now the distance values over non-tropical continental areas and oceans are similar.

In the continents the largest *d*_*i*_ values appear over the tropical rainforests of Africa and South America. These are regions dominated by monsoons and can be expected to have large *d*_*i*_ values because during the summer rainy season, when the insolation has its highest values, the solar energy is used mainly for evaporation instead of for heating. In the oceans there are coherent spatial structures, with high *d*_*i*_ values, in regions which tend to coincide with regions of deep convection in the Atlantic, Pacific and Indian oceans, including the Intertropical Convergence Zone (ITCZ). In these regions the SAT and rainfall are strongly coupled so that relatively small changes in SAT gradients modulate and shift the ITCZ. In particular, air-sea coupling in the eastern basins induce oceanic cold tongues which together with the continental geometry maintain warm waters and the ITCZ to the north of the equator. Thus, the high distance values in this region can be interpreted as being due to the strong air-sea coupling. Outside the 10°S–10°N band, the higher *d*_*i*_ values over the eastern subtropical north Pacific, as compared to the western basin, can be due to the influence of the semi-permanent anticyclone and associated stratus clouds which shield solar radiation. High latitude oceans (southern Ocean, Labrador sea, Greenland sea) also have relatively large *d*_*i*_ values, which can be explained by the existence of seasonal sea ice in the regions.

**Figure 2:**
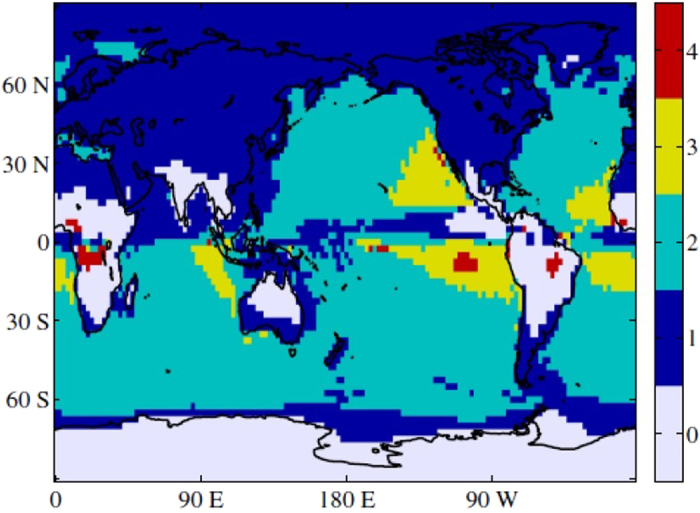
Map of *τ*_*i*_ values computed from ERA Interim reanalysis, with 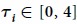. Matlab software (version number 7.12.0.635) was used to create these maps (https://www.mathworks.com/).

The map of *τ*_*i*_ values shown in Fig. 2 uncovers a rather symmetric behavior between both hemispheres. Overall, extratropical land masses have a lag of about 1 month, while extratropical oceans present a lag of 2 months, in agreement with McKinnon *et al*.^19^. In tropical oceanic areas *τ*_*i*_ displays values in the [0–4] range, with values close to 0 and 1 in the ITCZ region and *τ*_*i*_ = 3 in the eastern basins dominated by stratus clouds. Two continental regions, the African tropical rainforest and the Amazon rainforest, have a lag of 4 months, which could be due to the fact that in these regions SAT is colder in the summer compared to the spring because of intense rainfall.

**Figure 3:**
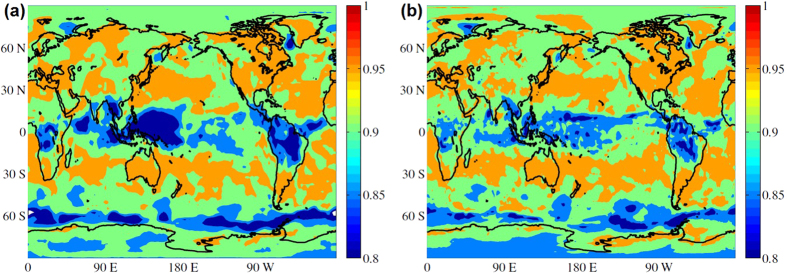
Shannon entropy of the SAT time series. (**a**) NCEP CDAS1 reanalysis, (**b**) ERA Interim reanalysis. Matlab software (version number 7.12.0.635) was used to create these maps (https://www.mathworks.com/).

Next, we present the results of the analysis of SAT unpredictability. Figure 3(a,b) display the entropy of SAT time series calculated from NCEP CDAS1 and ERA Interim reanalysis respectively. The main spatial patterns in the tropics resemble those in the distance map, Fig. 1(b), however, regions with large *d*_*i*_ values tend to have low entropy values. The linear correlation coefficient between *H*_*i*_ and *d*_*i*_ values is −0.45.

In NCEP CDAS1 reanalysis data, Fig. 3(a), there is a difference with ERA Interim reanalysis, Fig. 3(b), in the western Pacific. In this region, as will be discussed latter, the entropy values are relatively low because of the presence of extreme values (outliers) in the time series which render the PDF to be long-tailed.

**Figure 4:**
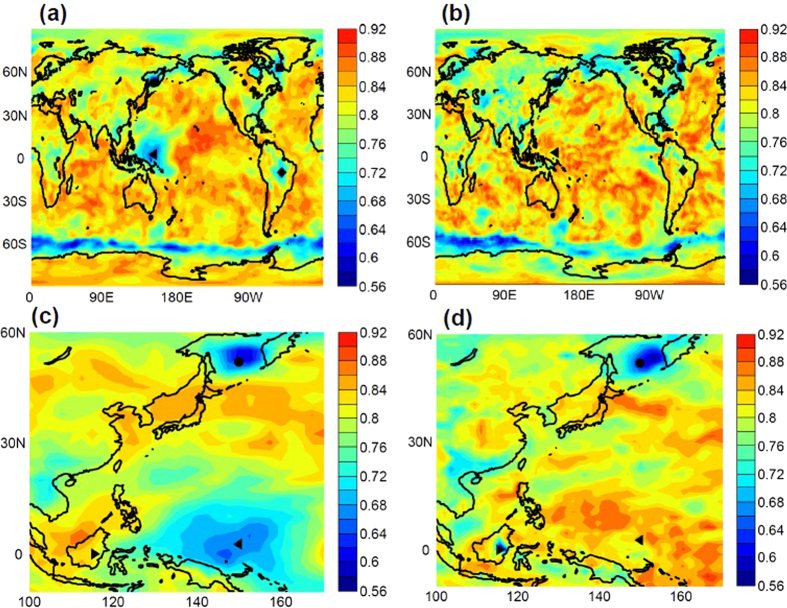
Map of Shannon entropy of SAT anomalies calculated from (**a**,**c**) NCEP CDAS1 reanalysis and from (**b**,**d**) ERA Interim reanalysis. The symbols indicate the geographical locations considered in Fig. 5: Sea of Okhotsk (northwest Pacific, indicated with a circle), Labrador Sea (square), Borneo (right triangle), western equatorial Pacific (left triangle) and the Amazonian region (diamond). Panels (c,d) show a detail of the western Pacific area: it can be observed that the two reanalysis have similar low entropy values in the region indicated with a circle, but have different entropy values in the regions indicated with left and right triangles. As shown in Fig. 5, the difference is due to the presence of extreme values in one dataset but not in the other. Matlab software (version number 7.12.0.635) was used to create these maps (https://www.mathworks.com/).

The entropy analysis of SATA time-series is presented in Fig. 4: panels (a) and (b) correspond to NCEP CDAS1 and ERA Interim reanalysis respectively, while panels (c) and (d) display a detail of the region where differences between the two datasets are found. In both SATA entropy maps we observe that the main spatial patterns in the tropical areas disappear and only those associated with sea ice remain.

**Figure 5:**
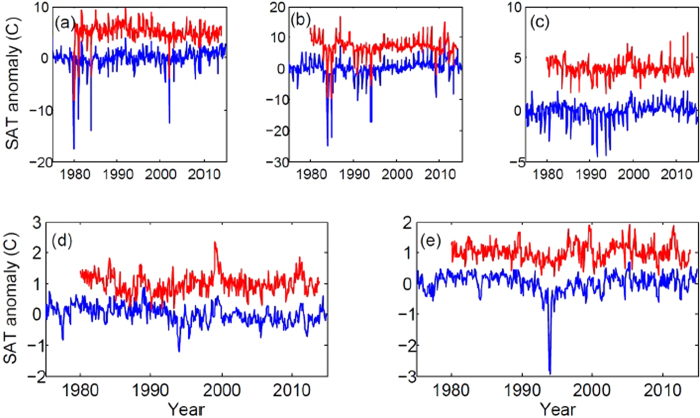
SAT anomaly time series from NCEP CDAS1 reanalysis (blue) and ERA Interim (red, displaced vertically by 1C for clarity). Panels (a–e) correspond to the geographical locations indicated in Fig. 4 with circle (Sea of Okhotsk, northwest Pacific), square (Labrador Sea), diamond (Amazonian region), right triangle (Borneo) and left triangle (western equatorial Pacific), respectively. In panels (d,e) some extreme values occur in one reanalysis but not in the other.

This observation can be explained by the fact that in regions associated with sea ice there is a strong seasonality in SAT variance that is not removed with the mean seasonal cycle. Examples of the SAT time series in these regions [indicated with a circle and with a square in Fig. 4(a,b)], are shown in Fig. 5(a,b). In these regions, the winter temperature can decrease considerably as the ice caps insulate the atmosphere from the ocean. In consequence, SATA PDF has a long tail in low temperature values, which is captured as a smaller entropy.

We also note that the map of the entropy computed from NCEP CDAS1 reanalysis is similar to that obtained from ERA Interim except in the western tropical Pacific where NCEP CDAS1 data shows lower entropy. A difference is also observed in the Amazon region, where NCEP CDAS1 data also has lower entropy. Figure 5(c–e) show the SATA time series in these regions, which are indicated with the symbols left triangle, right triangle and diamond in Fig. 4. We observe that there are some extreme values which occur in one reanalysis but not in the other.

## Discussion

In this section we compare our findings with results obtained within climate network approach. First, we can relate our findings with those of Hlinka *et al*.^9^ that presented a methodology for the identification and quantification of the non-linear contribution to the interaction information, able to identify main sources of nonlinearity in the nodes couplings. A comparison of the spatial structures uncovered in Fig. 1(b) here, with those in Fig. 5 9 (central panel), reveals that some of the areas with large distance values in Fig. 1(b) tend to coincide with the areas with nonlinear contribution to the mutual information. For example, the African and the Amazon rainforests are clearly seen in both maps. However, there are also differences: a ring in the Ocean around the Antarctica and a well defined region near the north pole (Greenland sea) that are strong signals in Fig. 5 9 are not strong in Fig. 1(b) here. However, in Fig. 4(b) here, we note that these regions are regions of low SAT anomaly entropy. Therefore, the regions with nonlinear contribution to mutual information seen in Fig. 5 9 are seen either in Fig. 1(b) or in Fig. 4(b) here. This observation suggests that the connectivity of these regions might reflect, in part, similar response to solar forcing, and/or similar SAT variability, with long-tailed distribution of SAT anomalies.

We can also relate our findings with those of Tirabassi and Masoller^7^, who analyzed the effects of lag-times in networks constructed from monthly SAT reanalysis. The maps of the lag-times between SAT time series in different regions, Fig. 2 7, for the three regions considered (Australia, El Niño basin and Mongolia) have a structure that is remarkably similar to the map presented in Fig. 2 here; however, a different procedure was used to compute the maps: the lag between any two regions was determined such as to superpose the two seasonal cycles (i.e., the lag was chosen to maximize the cross-correlation between the two SAT time series). In contrast, here the lag is determined by superposing the SAT time series and the insolation (by minimizing the distance between SAT time series and the insolation). The two approaches give several consistent observations. For example,

- In Fig. 2, right panel of ref. 7 the large red area in the North Hemisphere represents the geographical regions where the seasonal cycle is in-phase with that in Mongolia; comparing with Fig. 1(c) here we note that these are regions with a one month lag between the seasonal cycle and the isolation;
- In Fig. 2, left panel of ref. 7 the large red area in the Southern Hemisphere represents the regions where the seasonal cycle is in-phase with that in Australia; comparing with Fig. 1(c) here we note that in those regions, the lag between the seasonal cycle and the isolation is 0 or 1 month;
- In Fig. 2, center panel of ref. 7 the red area in the equator represents the regions where the seasonal cycle is in-phase with that in El Niño basin; comparing with Fig. 1(c) here we note that several well-defined structures appear in both maps, in particular the regions in yellow in Fig. 1(c), where the lag between the seasonal cycle and the isolation is 3 months, correspond to regions either in red or in light blue in Fig. 2, center panel of ref. 7.

The mutual lags between SAT time series in different regions were then used by Tirabassi and Masoller^13^ to infer *climate communities*, defined as regions that share similar properties of SAT time series. The map of the communities obtained, which are the regions which have a synchronous seasonal cycle, Fig. 1 13, has also several features in common with the map presented in Fig. 2 here. For example, the large communities formed by the oceans in the northern and in the southern hemispheres, represented with orange and blue in Fig. 1 13, have in Fig. 2 here a 2 month lag between SAT and the insolation. The similarity of these maps, despite the different way they were obtained, suggests that, at monthly scale, the mutual lag times are mainly determined by the seasonality induced by solar forcing.

The similarity of the ERA Interim maps and NCEP CDAS1 maps provides convincing evidence of the robustness of the results. The similarity is particularly remarkable considering that the datasets have different spatial resolution and temporal coverage. The different spatial resolution leads to more/less detail in the structures but has, in principle, no other influence in the results of the analysis. In contrast, some of the differences found when comparing the maps obtained from the two datasets could be attributed to their different temporal coverage.

A relevant problem is to estimate the uncertainty of the results. This could be done by using a subsampling approach; however, SAT/SATA non-stationarity may hamper the uncertainty estimation. An alternative approach is to perform the analysis using different runs of the same atmospheric general circulation model, with slightly different initial conditions. However, this study is out of the scope of the present work and is left for future work.

## Conclusions

In this work we have investigated the statistical properties of a climatological field (the surface air temperature, SAT) using two monthly reanalysis datasets: ERA Interim and NCEP CDAS1. We have quantified SAT unpredictability by means of Shannon entropy, *H*_*i*_, and we have analyzed the response to solar forcing, by means of a distance measure, *d*_*i*_, that assesses the similarity in the shape of the time series of the top-of-atmosphere incoming solar radiation (the insolation, *x*_*i*_) and the SAT, *y*_*i*_, both having been previously normalized to have zero mean and unit variance. A delay in the response of SAT to solar forcing was taken into account by lagging SAT with respect to the insolation.

We found that these two measures provide meaningful insight into global properties of SAT time series. In the distance map the tropics have considerable larger distance values, in comparison with the extratropics. There are well-defined structures in the oceans, over the Intertropical Convergence Zones, and over some continental areas, especially in regions largely dominated by monsoons, such as the tropical rainforest in Africa and South America, as well as over India. These regions do not respond to local insolation, but are characterized by strong air-sea coupling or land-atmosphere interaction which involve non-local processes.

In the SAT entropy maps we found qualitatively similar spatial structures, but with opposite high/low values: regions with high *d*_*i*_ values tend to correspond to regions of low entropy. When the entropy was calculated from SATA, the tropical spatial patterns disappeared but those in the high latitudes remained. This was interpreted as due to the fact that in high latitudes, mainly because of the presence of sea ice, there is a strong seasonality in variance that remains even when the annual cycle is removed.

While we found that the ERA Interim maps and the NCEP CDAS1 maps are remarkably similar, the entropy analysis also allowed to identify, in a well-defined region of the tropical western Pacific, relevant differences between the two datasets, which are likely due to the fact that there are some extreme values which occur in one reanalysis but not in the other. The different temporal coverage of ERA Interim and NCEP CDAS1 datasets might be another reason of these differences. A detailed comparison is, however, outside of the scope of the present work, which is aimed at demonstrating the suitability of two measures (the distance and the entropy) for quantifying similar properties of SAT time series in different regions.

It will be interesting, for future work, to compare the results presented here with those obtained by using other distance and entropy measures that lack the drawbacks of the measures used here, which were discussed in the Introduction. For computing the entropy, a promising approach for gaining more information is based in symbolic analysis^20^. In this approach a time series is first transformed into a sequence of symbols, and then, the entropy is computed from the probabilities of the symbols. In this case, depending on the specific rule employed to define the symbols, the entropy will capture different properties of the ordering of the values in the time series, and will give a different result when the data values are shuffled randomly. For computing the distance between SAT and insolation time series, several advanced approaches can be used, for example, each time-series can be mapped into a network (by using, e.g., recurrence^21^, visibility^22^, or symbolic networks^23^) and then, the dissimilarity of the two networks obtained can be computed^24^.

## Additional Information

**How to cite this article**: Arizmendi, F. *et al*. Identifying large-scale patterns of unpredictability and response to insolation in atmospheric data. *Sci. Rep.* **7**, 45676; doi: 10.1038/srep45676 (2017).

**Publisher's note:** Springer Nature remains neutral with regard to jurisdictional claims in published maps and institutional affiliations.

## Supplementary Material

Supplementary Information
