# Supplementary material for: Identifying large-scale patterns of unpredictability and response to insolation in atmospheric data
Source: Sci Rep. 2017 Mar 30;7:45676. doi: 10.1038/srep45676 (PMC5372476; doi:10.1038/srep45676)
Supplement: Supplementary Information [file srep45676-s1.pdf]

Supplementary information to *Identifying large-scale patterns of unpredictability and response to insolation in atmospheric data*

Fernando Arizmendi[†], Marcelo Barreiro[†], and Cristina Masoller[§*]
[†]*Instituto de Física, Facultad de Ciencias, Universidad de la República, Iguá 4225, Montevideo, Uruguay and*
[§]*Departament de Física, Universitat Politecnica de Catalunya, 08222 Terrassa, Barcelona, Spain*
(Dated: February 8, 2017)

We present a detailed analysis of the measure proposed in the main text to assess atmospheric response to solar forcing: we analyze the role of the interval in which we search for the minimum distance between time series, and we compare with an alternative measure. We also present a comparison of the results obtained from the two databases (NCEP CDAS1 and ERA Interim) and a comparison of the entropy maps computed with a uniform bin size.

In the main text we search for the minimum of the difference between two time-series, $x_i(t)$ and $y_i(t)$, Eq. (1) (reproduced here for convenience),

$$d_i = \sum_{t=1}^{L} |y_i(t) - x_i(t+\tau_i)|, \qquad (1)$$

with τ_i in the interval $[0, 4]$. Figure 1 displays the influence of the interval of τ_i values in which the minimum d_i is searched. From top to bottom these intervals are: $[0, 2]$, $[0, 3]$ and $[0, 5]$. We observe that similar spatial patterns are obtained, confirming the robustness of the methodology.

As discussed in the main text, if instead of using $|y_i(t) - x_i(t+\tau_i)|$ to define the difference, we use $(y_i(t) - x_i(t+\tau_i))^2$, then d_i is equal to $2(1-\rho_i)$ where ρ_i is the cross-correlation coefficient between $y_i(t)$ and $x_i(t+\tau_i)$. In Figs. 2(a) and (b) we compare the two approaches, and again we obtain very similar spatial patterns.

Figures 2(c) and (d) display the comparison of the distance maps obtained from the two datasets (NCEP CDAS1 and ERA Interim) and we observe a good qualitative agreement

Figure 3 displays the comparison of the entropy maps obtained from the two datasets. Different bin numbers are considered for computing the entropy. The two datasets give similar results except for well-localized regions, where extreme values occur as discussed in the main text. It is also observed that the patterns uncovered are robust with respect to the number of bins, M, used to compute the entropy.

Figures 4(a) and 4(b) display the standard deviation of the SATA time series for NCEP CDAS1 and ERA Interim respectively, while Figs. 4(c) and 4(d) display the corresponding entropy maps, computed by using a fixed bin size equal to $dy = (\max\{y\} - \min\{y\})/M$, where $\max\{y\}$ and $\min\{y\}$ are the maximum and minimum values in each dataset. One can notice that the entropy map computed in this way is similar to that of the standard deviation, except in the equatorial Pacific where the variance associated with the cold tongue stands out.

* e-mail to: arizmendi.f@gmail.com; e-mail to: cristina.masoller@upc.edu

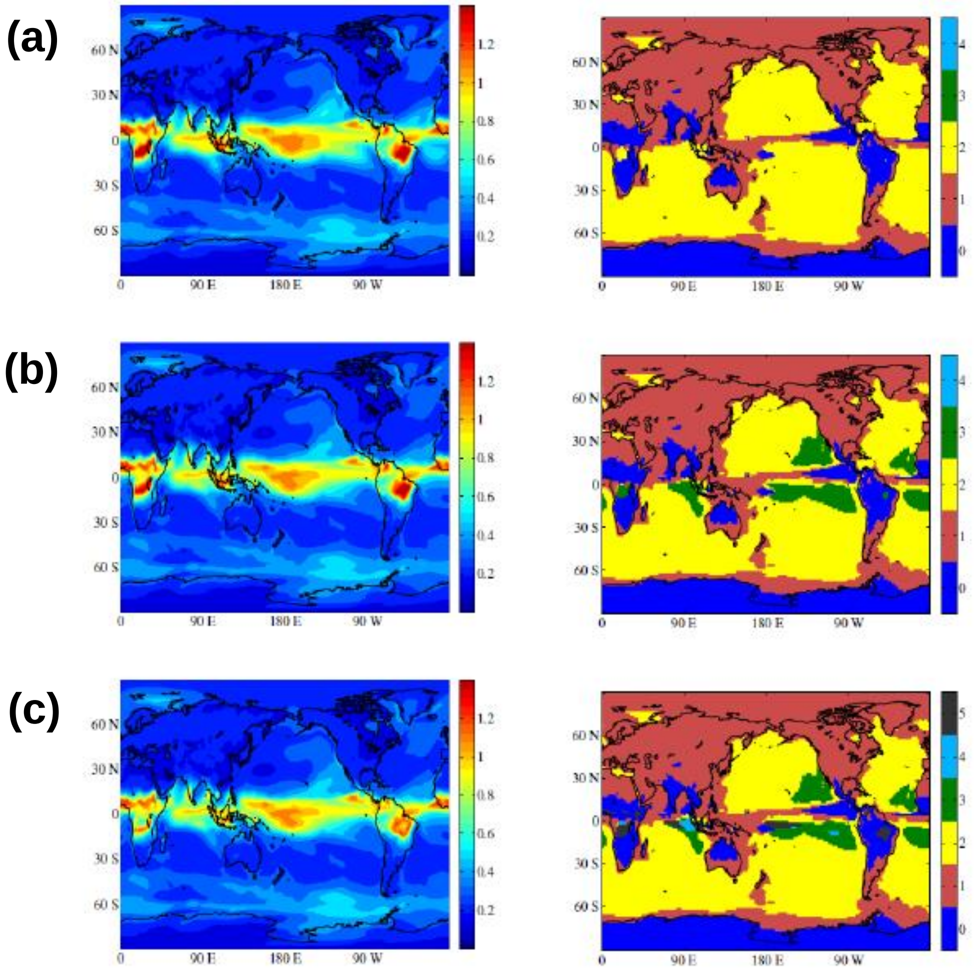

FIG. 1. Maps of distances d_i (left) calculated from Eq. (1) when the forcing and the response are shifted $\tau_i \in [0, 2]$ (a), $\tau_i \in [0, 3]$ (b) and $\tau_i \in [0, 5]$ (c). Maps of respective lags τ_i (right). Matlab software (version number 7.12.0.635) was used to create these maps (https://www.mathworks.com/).

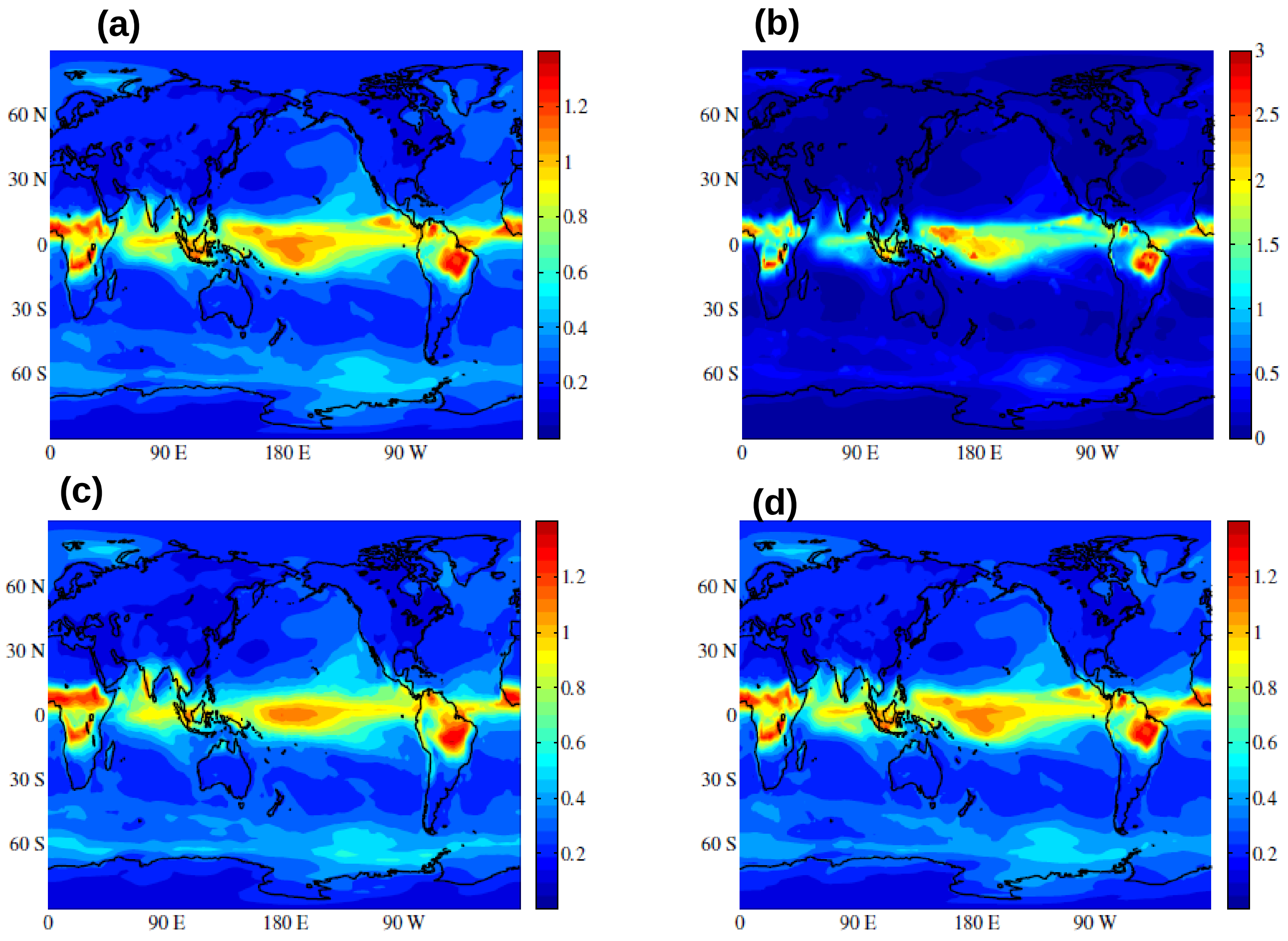

FIG. 2. Comparison between distance maps. In (a), d_i calculated from Eq. (1); in (b), d_i is calculated from Eq.(1), but $|y_i(t) - x_i(t+\tau_i)|$ is replaced by $(y_i(t) - x_i(t+\tau_i))^2$. In both cases the NCEP CDAS1 reanalysis is used and $\tau_i \in [0,4]$. (c), (d) Comparison between the distance maps obtained from NCEP CDAS1 (c) and ERA Interim (d) reanalysis. In both cases d_i calculated from Eq. (1) with $\tau_i \in [0,4]$. Matlab software (version number 7.12.0.635) was used to create these maps (https://www.mathworks.com/).

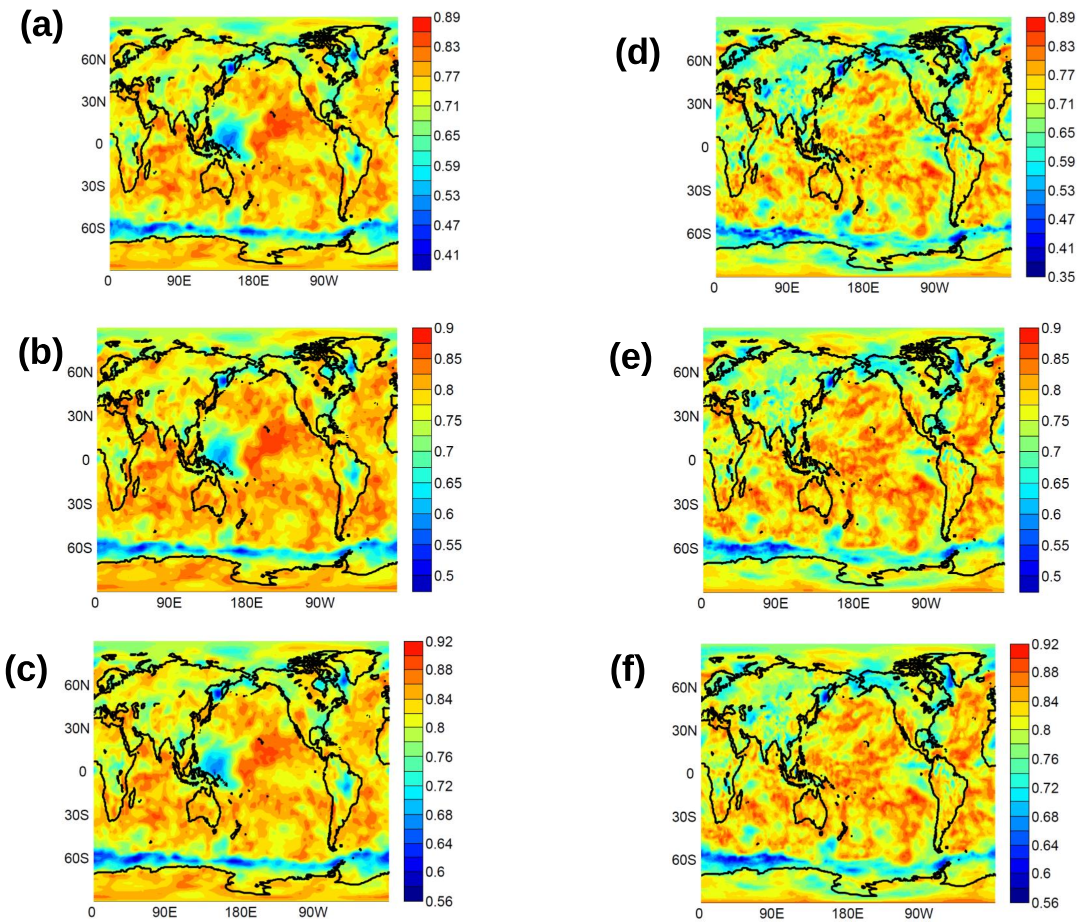

FIG. 3. Comparison of entropy maps obtained from NCEP CDAS1 (left) and ERA Interim (right). The number of bins used to compute the PDF of SAT anomalies is (a) 40, (b) 20, (c) 10, (d) 20, (e) 10 and (f) 5. Matlab software (version number 7.12.0.635) was used to create these maps (https://www.mathworks.com/).

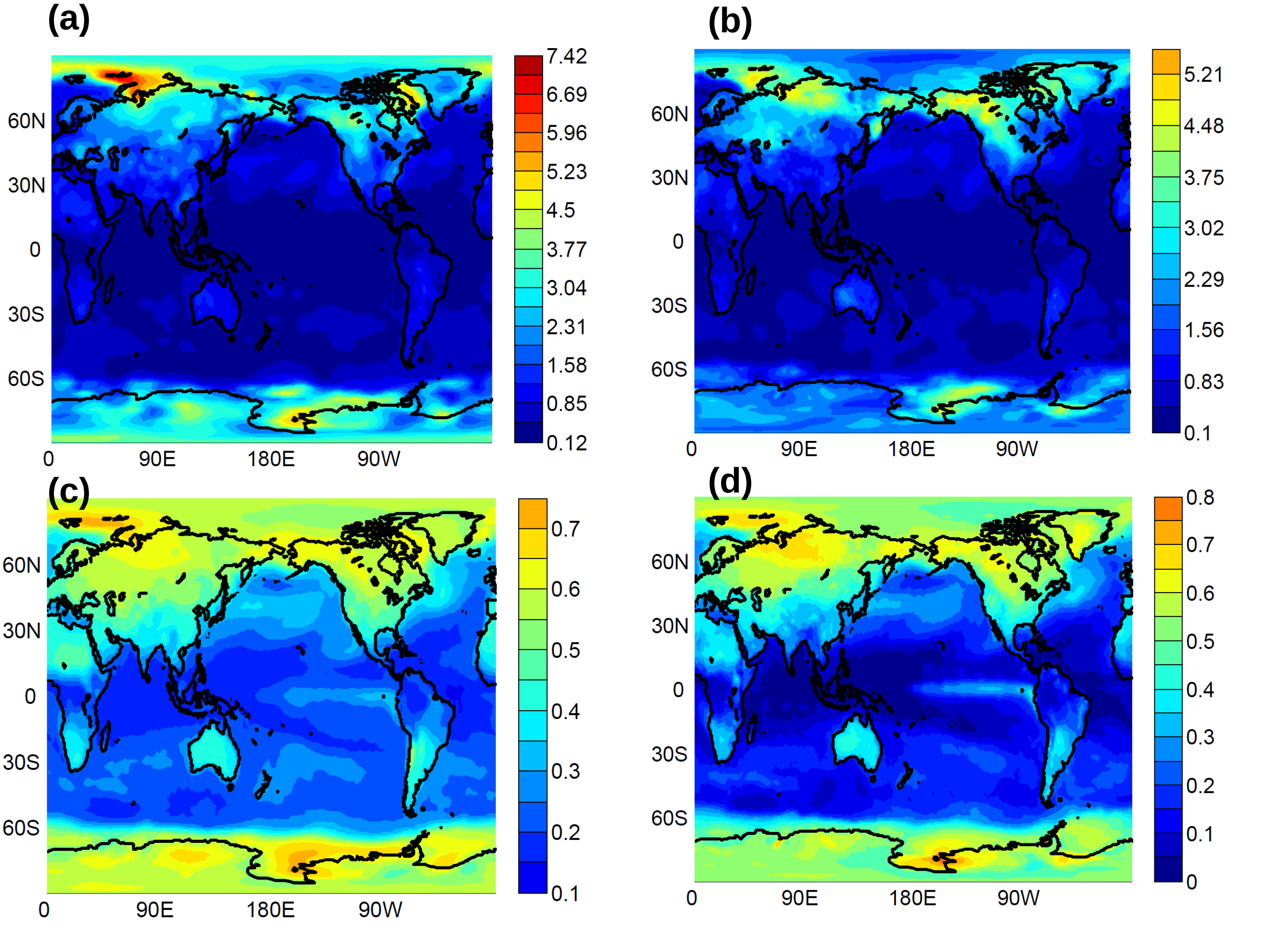

FIG. 4. Comparison of the maps of standard deviation of SAT anomalies obtained from NCEP CDAS1 (a) and ERA Interim (b). Entropy maps computed by using a uniform bin side defined from the global maximum and minimum values of the NCEP CDAS1 (c) and ERA Interim (d) datasets. Matlab software (version number 7.12.0.635) was used to create these maps (https://www.mathworks.com/).
